# A Geometric Modelling Approach to Determining the Best Sensing Coverage for 3-Dimensional Acoustic Target Tracking in Wireless Sensor Networks

**DOI:** 10.3390/s90906764

**Published:** 2009-08-27

**Authors:** Saeid Pashazadeh, Mohsen Sharifi

**Affiliations:** School of Computer Engineering, Iran University of Science and Technology, Tehran, Iran; E-Mail: msharifi@iust.ac.ir

**Keywords:** wireless sensor networks, 3-Dimensional acoustic target tracking, geometry and algebra, sensing coverage, square error

## Abstract

Existing 3-dimensional acoustic target tracking methods that use wired/wireless networked sensor nodes to track targets based on four sensing coverage do not always compute the feasible spatio-temporal information of target objects. To investigate this discrepancy in a formal setting, we propose a geometric model of the target tracking problem alongside its equivalent geometric dual model that is easier to solve. We then study and prove some properties of dual model by exploiting its relationship with algebra. Based on these properties, we propose a four coverage axis line method based on four sensing coverage and prove that four sensing coverage always yields two dual correct answers; usually one of them is infeasible. By showing that the feasible answer can be only sometimes identified by using a simple time test method such as the one proposed by ourselves, we prove that four sensing coverage fails to always yield the feasible spatio-temporal information of a target object. We further prove that five sensing coverage always gives the feasible position of a target object under certain conditions that are discussed in this paper. We propose three extensions to four coverage axis line method, namely, five coverage extent point method, five coverage extended axis lines method, and five coverage redundant axis lines method. Computation and time complexities of all four proposed methods are equal in the worst cases as well as on average being equal to *Θ*(1) each. Proposed methods and proved facts about capabilities of sensing coverage degree in this paper can be used in all other methods of acoustic target tracking like Bayesian filtering methods.

## 1. Introduction

3-dimensional acoustic target tracking is an extension of acoustic target tracking in 2-dimensional space in view of the fact that in real life applications we mostly deal with 3-dimensional target tracking. In target tracking temporal information of a target object in addition to its 3-dimensional spatial information must be computed. Target tracking in 3-dimensional space is a 4-dimensional problem and we consider time as the fourth dimension. The sensing information of each sensor node about a target object in its sensing coverage forms an equation. Simultaneous equations of target tracking are quadratic and non-linear therefore solving those using numerical methods is difficult, complex, and requires high computational resources that are generally constrained in the sensor nodes of wireless sensor networks (WSNs). In this paper we use geometry and algebra and exploit their relationship to model and prove some basic facts about 3-dimensional acoustic target tracking and propose new methods for computing the spatio-temporal information of a target object. Our proposed methods use linear simultaneous equations instead of quadratic equations and need less computational resources, making them more amenable to target tracking applications with timing constraints. We prove that four sensing coverage of a target object cannot always yield the correct spatio-temporal information of a target object and that this proven fact is quite independent of the kind of used target tracking method.

In this paper we show that simultaneous equations of four sensing coverage for a target object yield two different answers. Most of the times we can eliminate the infeasible answer by performing a simple proposed time test. We introduce a four coverage axis line method which works based on the four sensing coverage. We prove that the sensing information of five sensor nodes about a target object accurately determine the correct spatio-temporal information of the target object. To overcome this weakness, we propose to increase the sensing coverage to five sensing nodes and present three new methods as extensions to the four coverage axis line method. Five coverage extent point method uses the sensing information of five sensor nodes and by solving a set of four linear equations accurately determines the spatio-temporal information of a target object. Five coverage extended axis line method is based on the four coverage axis line method and if the sensing information of four sensor nodes about a target object does not satisfy conditions to remove the infeasible answer out of two answers of the set of its simultaneous linear equations, the sensing information of a fifth sensing node on the same target object is used to determine the correct spatio-temporal information of the target object. Our last proposed method called five coverage redundant axis line method is based on the five sensing coverage but uses the set of simultaneous equations of four sensing coverage in at least two different sets. This method deploys a customized version of formal majority voter among sensory nodes to compute the spatio-temporal information of a target object. The contributions of our paper are applicable to other methods of target tracking like Bayesian filtering, Kalman filtering [1], and Particle filtering [2]. All analytic discussions and presented algorithms in this paper are applicable to both wired and wireless platforms of sensor nodes. For the sake of brevity, simulations are only performed for a wireless sensor nodes platform. Proposed theorems and methods are equally applicable to time of flight radio positioning systems. In these systems beyond the acoustic signals we can use radio signals or other types of signals for tracking the target object. The main discussion of this paper is about the target localization in acoustic target tracking.

The rest of this paper is organized as follows. Section 2 presents related work in the area of 3-dimensional acoustic target tracking. Section 3 presents the basics of 3-dimensional acoustic target tracking and geometric representation of problem and its equivalent dual geometric problem that can be solved easier than the main problem. Section 4 discusses theoretically the special properties of dual representations of 3-dimensional acoustic target localization and introduces the theoretical basis for a method based on the four sensing coverage. Section 5 introduces a four coverage axis line method based on the theoretic background of Section 4 and analyses the simulation results of its application to a real life problem. Section 6 theoretically proves that five sensing coverage always guarantees to yield the correct answer. It also presents three extended methods based on the four coverage axis line method in Section 5, alongside the necessary theorems and proofs and simulation results in support of these methods. Section 7 concludes the paper and suggests some future work.

## 2. Related Work

A considerable part of the literature on WSNs discusses the issues of sensor node localization and location tracking [3,4]. Some node localization approaches measure the time difference of RF signal propagations to compute the distance between sensor nodes. Other approaches use the signal strength to measure the distance between sensor nodes for sensor node localization [3,4]. Trilateration and multilateration techniques are used for sensor node localization [3,4]. Target localization using sound detection has similar simultaneous equations with sensor node localization; but target localization equations contain an extra temporal unknown variable that makes them different from sensor node localization. Wang *et al*. [5] studied the basics of acoustic target tracking and showed the possibility of using this method to accurately track targets using WSNs. They used quality rank to target tracking result and quality-driven redundancy suppression and contention resolution to improve the information throughput. Gupta and Das [6] discuss several factors that influence the accuracy of target tracking and their potential problems. The accuracy of their results is however greatly influenced by the number of location estimation samples. Their studies had higher error margin than it is possible to be applicable to real applications.

Brooks *et al*. [7] studied the basics of tracking single and multiple targets that are sufficiently separated in space and/or time. They have used a technique called lateral inhibition to reduce the computational and network costs while maintaining an accurate tracking. Lin *et al*. [8] formulated the object tracking problem as an optimization problem. They presented two message-pruning structures for tracking moving objects, taking into account the physical topology of the network to reflect the real communication costs [8].

Ekman *et al*. [9] used Bayesian framework for acoustic target tracking by developing particle filters that use data association techniques based on probabilistic data associations. Simultaneous localization, tracking and calibration based on Bayesian filter are studied in two and three dimensional indoor and outdoor spaces with accurate results [10,11]. Special types of Kalman filtering [12] are used to overcome some of the problems of acoustic target tracking such as the problem of global time synchronization.

Using WSNs for real-time target tracking with guaranteed deadlines had been studied by He *et al*. [13]. They studied relations between sensor density, velocity of a moving target and wake-up delay of sensor nodes under real-time constraints. Distributed data associations of sensors’ measurements are used in multiple targets tracking [14]. Decentralized dynamic clustering for target tracking is an approach that is derived and evaluated by some researches [15]. Using acoustic signal energy measurements of individual sensor nodes to estimate the locations of multiple acoustic sources is another approach that is used for target tracking [16]. Studies show that the maximum likelihood acoustic source location estimation method compared to existing acoustic energy based source localization methods yields more accurate results and enhances the capability of multiple source localization.

Barsanti *et al*. [17] studied the tracking of objects with constant velocity in uncertain locations, in addition to studying various scenarios related to locating sensor nodes. Good time synchronization and localization accuracy are reported as the essential prerequisites for accurate target tracking [18]. Using measured time difference of arrival (TDOA) for a sensor array for joint estimation of source location and propagation speed is done using two different techniques [18]. Dan *et al*. [19] proposed collaborative signal processing (CSP) as a framework for tracking multiple targets in a distributed WSN. The key components include event detection, estimation and prediction of target location, and target classification. Chuang [20] classified target tracking approaches from another viewpoint to three categories: tree-based, cluster-based, and prediction-based.

Combining geometry and algebra to represent the spatio-temporal information of target objects is a new idea. We had studied the geometric modeling of 2-dimensional acoustic target tracking using wireless sensor nodes and proved that three sensing coverage can only sometimes determine the correct spatio-temporal information of a target object in 2-dimensional acoustic target tracking. We proved that four sensing coverage is the best sensing coverage for 2-dimensional target tracking and proposed new geometric methods with low computational overhead [21]. The current paper is the extension of our previous work [21] on 3-dimensional acoustic target tracking.

## 3. Basics of 3-Dimensional Acoustic Target Tracking

### 3.1. 3-Dimensional Acoustic Target Localization Model

In this paper we have ignored the signal processing aspects of acoustic target tracking and assumed that the sound waves of a target object is detected and differentiated from other environmental sounds by an appropriate signal processing method. We have also ignored the environmental phenomena that may affect the broadcasting of sound waves in 3-dimensional space and the reflections of sound waves when they meet the ground surface. Furthermore, we have assumed that every sensor node is equipped with a microphone for sensing sound waves, and localization and time synchronization of all sensor nodes are done with high accuracy.

**Figure 1.**
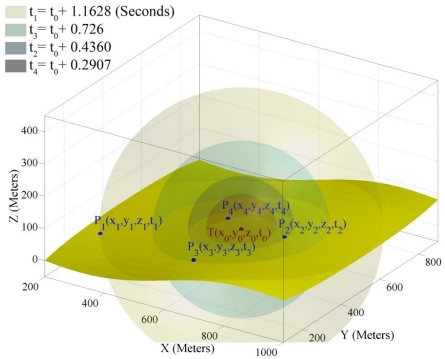
Basic schema of target localization in 3-dimensional space.

When a target object in an unknown location (*x*_0_, *y*_0_, *z*_0_) generates sound waves at time *t*_0_, its sound waves broadcast in a spherical form in 3-dimensional space and reaches to each sensor node in the field after some time delay that directly depends on the Euclidian distance of sensor nodes from the target object. Figure 1 shows the basic schema of acoustic target localization in 3-dimensional space by sensing sound waves of a moving target object. When a sensor node *P_i_* senses a sound wave and detects that this sound belongs to a target object of interest, it generates a record consisting of four fields in the form of (*x_i_*, *y_i_*, *z_i_*, *t_i_*). The first three fields represent the 3-dimensional coordinates of the sensing node and the fourth field represents the time at which this mote has sensed the sound of the target object. Each mote broadcasts this record to its neighboring motes in its communication range. The goal is to cooperatively compute the four unknown variables (*x*_0_, *y*_0_, *z*_0_, *t*_0_) that represent the spatio-temporal information of target object in a distributed way. This information implies that the target object has generated a sound at time *t*_0_ in position (*x*_0_, *y*_0_, *z*_0_) that has been detected by motes after some delay. To compute these four unknown variables, we need the sensing information of at least four sensor nodes to create simultaneous equations that contain at least four equations.

Let us assume that sound waves propagate with constant speed of v = 344.0 m/s. Based on simple formulas of physics for displacement with constant velocity Δ*x* = *ν·Δt* relation, the distance Δ*x* of a sensor node from a target object is equal to sound propagation speed (*v*) multiplied by the time delay (Δ*t*). Time delay is equal to the difference between the sensing sounds of the target object by a sensor node and the sound generation time of the target object. The sensing information of four sensor nodes gives us the simultaneous equations of target localization as follows:

(1) $$\{\begin{array}{l} {\left(x-x_{1}\right)}^{2}+{\left(y-y_{1}\right)}^{2}+{\left(z-z_{1}\right)}^{2}={\left(t_{1}-t\right)}^{2}\;v^{2}\\ {\left(x-x_{2}\right)}^{2}+{\left(y-y_{2}\right)}^{2}+{\left(z-z_{2}\right)}^{2}={\left(t_{2}-t\right)}^{2}\;v^{2}\\ {\left(x-x_{3}\right)}^{2}+{\left(y-y_{3}\right)}^{2}+{\left(z-z_{3}\right)}^{2}={\left(t_{3}-t\right)}^{2}\;v^{2}\\ {\left(x-x_{4}\right)}^{2}+{\left(y-y_{4}\right)}^{2}+{\left(z-z_{4}\right)}^{2}={\left(t_{4}-t\right)}^{2}\;v^{2} \end{array}$$

All equations in (1) are degree two. Solving this set of simultaneous equations using iterative numerical methods like Newton method is computationally intensive. We use geometric modeling to find an equivalent set of simultaneous equations that can be solved with less computation.

### 3.2. Geometric Representation of 3-Dimensional Acoustic Target Localization

The general equation of a right spherical double hypercone (spherical cone) in 4-dimensional space whose apex point has (*x*_0_, *y*_0_, *z*_0_, *t*_0_) coordinates is as follows [22]:

(2) $$\frac{{\left(X-x_{0}\right)}^{2}+{\left(Y-y_{0}\right)}^{2}+{\left(Z-z_{0}\right)}^{2}}{a^{2}}=\frac{{\left(T-t_{0}\right)}^{2}}{c^{2}}$$

Capital letters *X*, *Y*, *Z*, and *T* in this equation represent free variables. One famous method for visualizing the shape of a 4-dimensional object is to map it to a 3-dimensional space. In this paper we consider the fourth dimension as time and keep it fixed and draw the 3-dimensional shape of the object at fixed points in time. If we serialize the different 3-dimensional images of the object in a time period, we can imagine the 4-dimensional shape of the object [23].

**Figure 2.**
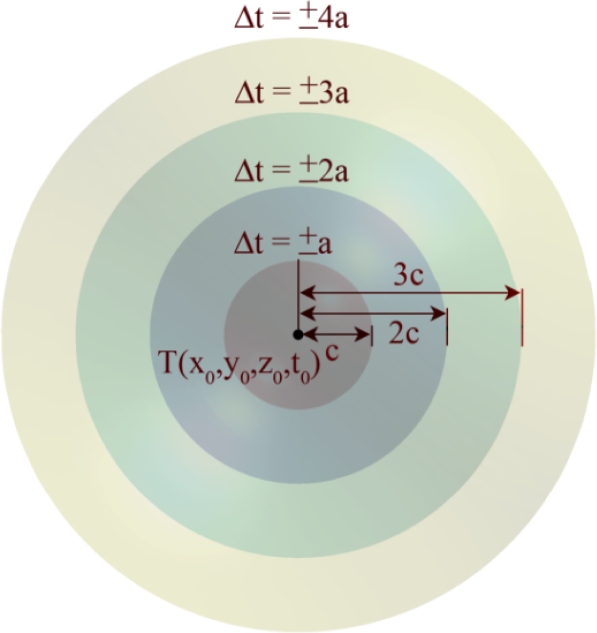
A general right spherical double hypercone in 4-dimensional space mapped to 3-dimensional space at five different points in time.

If the *T* coordinate of the equation of the right spherical double hypercone in Equation (2) is interpreted as time, then the shape of it will be like the sphere at different points in time. Figure 2 shows the 3-dimensional spatial shape of a 4-dimensional unlimited right spherical double hypercone of degree two Equation (2) at five different points in temporal dimension. The 4-dimensional apex point of this cone is (*x*_0_, *y*_0_, *z*_0_, *t*_0_) point. Shape grows in spherical form centered at the apex point with the ratio of *a*/*c* with respect to the fourth (time) dimension.

When time increases (up nappe) or decreases (down nappe) relative to *t*_0_, the 3-dimensional shape of Equation (2) becomes an increasing radius sphere as it is shown in Figure 2. The upper nappe is called *the future hypercone* and the lower nappe is called *the past hypercone.* Equation (2) can be converted to the equation of sound propagation in 3-dimensional space with respect to time by assuming *a* = *v* = 344.0 (m/s) and *c* = 1 making it in the form of equations in Equation (1).

By looking at simultaneous equations in Equation (1) we can assume that the sensing information of each sensor node *P_i_*(*x_i_*, *y_i_*, *z_i_*, *t_i_*) is a 4-dimensional point from surface of up nappe of double hypercone defined in Equation (2). If we substitute the coordinates of each sensing node in Equation (2), we derive the simultaneous equations of Equation (1). Therefore, the geometric representation of the target localization problem can be stated as: *finding the four dimensional coordinates of the apex point* (*x*, *y*, *z*, *t*). *of the right spherical double hypercone using four known points P_i_ on the surface of the up nappe.* Because sound can be sensed only after its production, the sensing information of sensor nodes are related to the up nappe.

### 3.3. Dual Geometric Representation of Acoustic Target Localization

We represented the 3-dimensional acoustic target localization problem as a geometric problem in previous section and now we want to present an equivalent dual representation of this problem from another viewpoint. Answers of these two dual interpretations are the same because both of them use the same set of Equation (1). Solving dual geometric representation of the problem is easier and more straightforward than that in the general form.

Each equation in simultaneous equations of Equation (1) represents a sound propagation hypercone in 4-dimensional space whose apex point is *P_i_* (*x_i_*, *y_i_*, *z_i_*, *t_i_*), where *i* = 1,2,3,4. So, the sensing information of each sensor node *i* is a 4-dimensional double hypercone whose apex point is the sensing information of that sensor node. The target localization problem can thus be redefined from this new viewpoint as: *finding the 4-dimensional coordinates of the point* (*x*, *y*, *z*, *t*) *that lie on all 4-dimensional sound propagation double hypercones whose apex points are sensing nodes’ information.* Hereafter in the paper we solve the dual representation of the 3-dimensional acoustic target localization problem.

## 4. Combined Algebraic and Geometric Solution to 3-Dimensional Acoustic Target Localization

### 4.1. Geometric Properties of Two Sensor Node’s Information

***Definition 1.*** Sensing information of each sensor node *i* represents a 4-dimensional right spherical double hypercone that we call it *the sensing hypercone*. We denote the sensing hypercone of a sensor node *i* by *η_i_* and its equation as follows:

(3) $$\eta_{i}:{\left(x-x_{i}\right)}^{2}+{\left(y-y_{i}\right)}^{2}+{\left(z-z_{i}\right)}^{2}-{\left(t_{i}-t\right)}^{2}\;v^{2}=0$$

*Affine hyperplane* or just *hyperplane* in 4-dimensional space (ℝ^4^) is a 3-dimensional subspace of ℝ^4^ whose equation is as follows [24]:

(4) Ax+By+Cz+Dt+E=0

Hereafter in this paper, hyperplane is taken synonymous to hyperplane in 4-dimensional space.

***Lemma 1.*** The intersection of two different sensing hypercones resides on a hyperplane.

***Proof.*** If a point is a solution to both sensing hypercones (the intersection) then it is also a solution to any linear combinations of both sensing hypercones’ equations. By subtracting the sensing hypercone of a sensor node *j* from the sensing hypercone of a sensor node *i*, the following equation is derived:

(5) $$\begin{array}{c} \left({\left(x-x_{i}\right)}^{2}+{\left(y-y_{i}\right)}^{2}+{\left(z-z_{i}\right)}^{2}-{\left(t_{i}-t\right)}^{2}\;v^{2}\right)-({\left(x-x_{j}\right)}^{2}+{\left(y-y_{j}\right)}^{2}\\ +{\left(z-z_{j}\right)}^{2}-{\left(t_{j}-t\right)}^{2}\;v^{2})=0 \end{array}$$

that can be simplified to:

(6) $$\begin{array}{ll} 2\left(x_{j}-x_{i}\right)x+ & 2\left(y_{j}-y_{i}\right)y+2\left(z_{j}-z_{i}\right)z+2\left(t_{i}-t_{j}\right)v^{2}t\\ & \;+\left(\left(x_{i}{}^{2}-x_{j}{}^{2}\right)+\left(y_{i}{}^{2}-y_{j}{}^{2}\right)+\left(z_{i}{}^{2}-z_{j}{}^{2}\right)+\left(t_{j}{}^{2}-t_{i}{}^{2}\right)v^{2}\right)=0 \end{array}$$

Equation (6) is the general form of a hyperplane’s equation as it is shown in Equation (4). This shows that differencing the equations of two sensing hypercones in the given form is a hyperplane and thus any intersection point of the two hypercones also lies on this hyperplane. This means that the intersection of each pair of sensing hypercones of simultaneous equations of target localization is a surface that lies in a hyperplane in ℝ^4^.

***Definition 2.*** The intersection of two sensing hypercones resides on a hyperplane we call it *the intersection hyperplane*. We denote the intersection hyperplane made by the sensing hypercones of sensor nodes *i* and *j* by *π_ij_* as in the following equation:

(7) $$\begin{array}{c} \pi_{\mathrm{ij}}:A_{\mathrm{ij}}\;x+B_{\mathrm{ij}}\;y+C_{\mathrm{ij}}\;z+D_{\mathrm{ij}}\;t+E_{\mathrm{ij}}=0\\ A_{\mathrm{ij}}=2\left(x_{j}-x_{i}\right),\;B_{\mathrm{ij}}=2\left(y_{j}-y_{i}\right),\;C_{\mathrm{ij}}=2\left(z_{j}-z_{i}\right),\;D_{\mathrm{ij}}=2\left(t_{i}-t_{j}\right)v^{2},\\ E_{\mathrm{ij}}=\left(\left(x_{i}{}^{2}-x_{j}{}^{2}\right)+\left(y_{i}{}^{2}-y_{j}{}^{2}\right)+\left(z_{i}{}^{2}-z_{j}{}^{2}\right)+\left(t_{j}{}^{2}-t_{i}{}^{2}\right)v^{2}\right) \end{array}$$

***Definition 3.*** A pair of sensing hypercones *η_i_* and *η_j_* intersect on a 4-dimensional degree two surface that we denote by *σ_ij_*.This surface resides on the hyperplane *π_ij_* and we call it *the intersection surface* of two sensing hypercones.

### 4.2. Demonstrating Geometric Properties of Two Sensor Node’s Information

**Figure 3.**
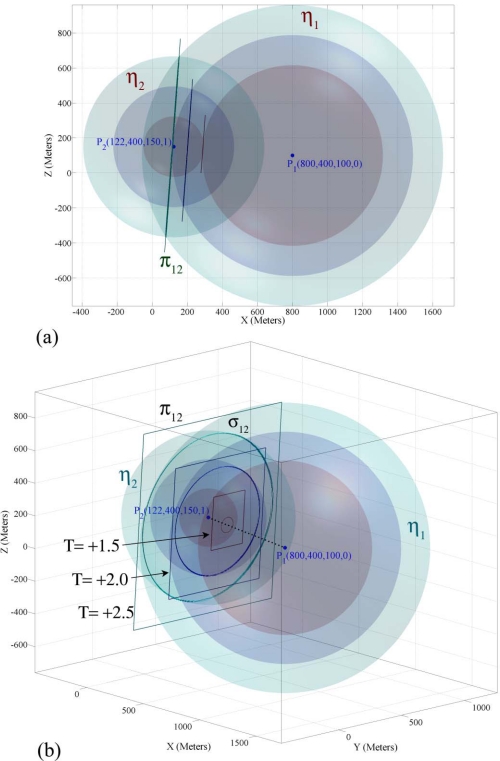
(a) The intersection hyperplanes of two double hypercones at three different points in time. (b) The intersection of two 4-dimensional double hypercones of target localization in 3-dimensional space.

To clarify the results of lemmas and theorems, we use an example 3-dimensional target localization problem. We assume that a target object at location *T* (670, 604, 10) has generated a sound wave at time 0.0 and four sensor nodes have sensed its sound waves. The sensing information of the four sensor nodes are *P*_1_ (800, 400, 100, 0.75029), *P*_2_ (122, 400, 150, 1.7479), *P*_3_ (400, 800, 200, 1.1161), and *P*_4_ (500, 200, 125, 1.3173). Figure 3a shows two 4-dimensional sensing hypercones *η*_1_ and *η*_2_ alongside their intersection hyperplanes π_12_ in three different points in time. Figure 3b shows these two sensing hypercones from another viewpoint and also shows the intersection surface of them in three different points in time. In visualization of the 4-dimensional shapes mapped to 3-dimensional space shown in Figure 3, the hypercones are shown as spheres and hyperplanes as planes at different points in time. Every section of a sphere and a plane is a circle and the intersection curve of two spheres is a circle too [25]. If two spheres intersect, their intersection will be a circle that resides on a 2-dimensional plane. In visualization of 4-dimensional shapes in Figure 3, we see that each pair of unbounded 4-dimensional sensing hypercones intersects on a 4-dimensional curve that resides on their intersection hyperplane.

Based on Lemma 1, we can now compute the intersection surface of two sensing hypercones more easily by computing the intersection of each sensing hypercone with their common intersection hyperplane. To introduce intersection surface of two sensing hypercones, we need to define hyperconic sections.

**Figure 4.**
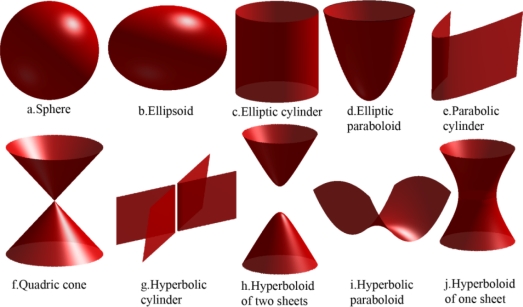
Possible quadric surfaces built from the intersection of a hypercone with a hyperplane [30].

The *conic sections* are the curves generated by the intersections of a plane with one or two nappes of a double circular right cone in (ℝ^3^). Circle, ellipse, parabola, and hyperbola are four different quadratic curves that can be produced from the intersection of a cone and a plane [26–28]. We use *hyperconic sections* in our study that are the extension of the conic section to 4-dimensional space. A hyperplane intersects with a right spherical double hypercone in a quadric surface [29]. Figure 4 shows ten different possible quadric surfaces that can be produced from the intersection of a hyperplane with a right circular spherical double hypercone in 4-dimensional space [30,31].

**Figure 5.**
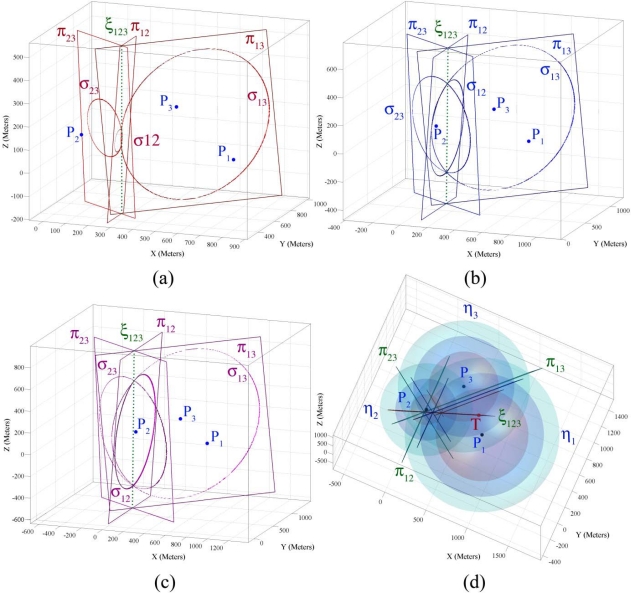
The intersection surfaces, the pencil and the axis plane of three sensing hypercones (a). At time t = +1.5, (b). At time t = +2.0, and (c). At time t = + 2.5, (d). The axis plane of the pencil passes through point that representing real target object’s spatio-temporal information.

The intersection of each pair of sensing hypercones is a hyperconic section that is mostly in the form of a hyperboloid of two sheets or an elliptic paraboloid. All sensing hypercones that we deal with in target localization in this paper are right circular hypercones with equal aperture angles whose axis are parallel. That is why the intersection hyperplanes of sensing hypercones do not have big angles with the axis line of hypercones, implying that their intersection surface will not be in the form of some of the hyperconic sections like sphere and ellipsoid. The intersection surface of two hypercones in Figure 3 and Figure 5 is a hyperboloid of two sheets, but in these figures you can see only half of a hyperboloid of two sheets; because we have only drawn the part of figure that is related to the future time.

### 4.3. An Algebraic Representation of 3-Dimensional Acoustic Target Localization

We use the relation of linear algebra with geometry to study the properties of intersection hyperplanes in ℝ^4^. We can represent the equations of intersection hyperplanes of each pair of sensing hypercones in the form of *system of linear equations* [32]. The dimension of the solution space of a homogeneous system of linear equations *AX =* 0 is *n−r* where *n* is the number of unknowns and *r* is the rank of the coefficient matrix *A* that is the number of maximally linearly independent rows of matrix *A* [32–34]. Equation *AX = B* has a solution if and only if *rank A = rank* [*A B*] that means the ranks of the coefficient matrix and the augmented *matrix [A B*] be equal [32,35].

***Theorem 1.*** Two non parallel hyperplanes always intersect in a plane.

***Proof.*** If two hyperplanes in 4-dimensional space are not parallel, then their normal vectors are linearly independent. Simultaneous linear equations of such two hyperplanes will have dimension 4 − 2 = 2. Therefore, the intersection of two non parallel hyperplanes in 4-dimensional space is a plane.

### 4.4. Geometric Properties of Three Sensor Node’s Information

Three sensing hypercones can have three different paired combinations and thus have three intersection hyperplanes. In this part we study the geometric properties of three sensing information.

***Definition 4.*** All planes in ℝ^3^ that pass from a common straight line form a *pencil* and the common straight line is called *the axis of pencil* [36].

Now we extend the definition of pencil to ℝ^4^

***Definition 5.*** All hyperplanes in ℝ^4^ that pass through a common plane form a *pencil of hyperplanes*. Any two of these hyperplanes may be used to define the intersection plane [37]. We name the axis of the pencil that is constructed by the intersection hyperplanes of sensing hypercones *η_i_*, *η_j_* and *η_k_* make, as *the axis plane* and denote it by *ξ_ijk_*.

***Lemma 2.*** Let us assume that equations of two independent hyperplanes are as follows:

(8) $$\{\begin{array}{l} A_{1}\;x+B_{1}\;y+C_{1}\;z+D_{1}\;t+E_{1}=0\\ A_{2}\;x+B_{2}\;y+C_{2}\;z+D_{2}\;t+E_{2}=0 \end{array}$$

If a third hyperplane’s equation satisfies the following condition:

(9) $$\begin{array}{ll} A_{3}\;x+B_{3}\;y+ & C_{3}\;z+D_{3}\;t+E_{3}\\ & =k_{1}\left(A_{1}\;x+B_{1}\;y+C_{1}\;z+D_{1}\;t+E_{1}\right)+k_{2}\left(A_{2}\;x+B_{2}\;y+C_{2}\;z+D_{2}\;t+E_{2}\right)\\ & =0 \end{array}$$

where *k*_1_,*k*_2_ ∈ ℝ, … then three hyperplanes make a pencil.

***Proof.*** Let us consider the equation of three hyperplanes as a set of simultaneous equations. If condition of Equation (9) holds, it implies that the equation of the third hyperplane is a linear combination of equations of two first hyperplanes. Therefore the coefficients of only two hyperplanes are linearly independent and the rank of the coefficients will be two and we have four unknown parameters. Dimension of result will be 2 and these three hyperplanes intersect with each other on a common plane and make a pencil.

***Theorem 2.*** Intersection hyperplanes of three sensing hypercones make a pencil.

***Proof.*** Let us assume that three sensing nodes are *i*, *j*, *k* and based on Definition 2 their paired sensing hypercones makes intersection hyperplanes as *π_ij_*, *π_ik_*, *π_jk_*. In the following equation the first equation represents the intersection hyperplane *π_ij_* and the second equation represents the intersection hyperplane *π_ik_*:

(10) $$\{\begin{array}{l} \pi_{\mathrm{ij}}:2\left(x_{j}-x_{i}\right)x+2\left(y_{j}-y_{i}\right)y+2\left(z_{j}-z_{i}\right)z+2\left(t_{i}-t_{j}\right)v^{2}t+\\ \left(\left(x_{i}{}^{2}-x_{j}{}^{2}\right)+\left(y_{i}{}^{2}-y_{j}{}^{2}\right)+\left(z_{i}{}^{2}-z_{j}{}^{2}\right)+\left(t_{j}{}^{2}-t_{i}{}^{2}\right)v^{2}\right)=0\\ \pi_{\mathrm{ik}}:2\left(x_{k}-x_{i}\right)x+2\left(y_{k}-y_{i}\right)y+2\left(z_{k}-z_{i}\right)z+2\left(t_{i}-t_{k}\right)v^{2}\;t+\\ \left(\left(x_{i}{}^{2}-x_{k}{}^{2}\right)+\left(y_{i}{}^{2}-y_{k}{}^{2}\right)+\left(z_{i}{}^{2}-z_{k}{}^{2}\right)+\left(t_{k}{}^{2}-t_{i}{}^{2}\right)v^{2}\right)=0 \end{array}$$

If we substitute the equations of hyperplanes *π_ij_*, and *π_ik_* from Equation (10) in Equation (9), the following equation holds:

(11) $$\begin{array}{c} k_{1}(2\left(x_{j}-x_{i}\right)x+2\left(y_{j}-y_{i}\right)y+2\left(z_{j}-z_{i}\right)z+2\left(t_{i}-t_{j}\right)v^{2}\;t+\\ \left(\left(x_{i}{}^{2}-x_{j}{}^{2}\right)+\left(y_{i}{}^{2}-y_{j}{}^{2}\right)+\left(z_{i}{}^{2}-z_{j}{}^{2}\right)+\left(t_{j}{}^{2}-t_{i}{}^{2}\right)v^{2}\right))+\\ k_{2}(2\left(x_{k}-x_{i}\right)x+2\left(y_{k}-y_{i}\right)y+2\left(z_{k}-z_{i}\right)z+2\left(t_{i}-t_{k}\right)v^{2}t+\\ \left(\left(x_{i}{}^{2}-x_{k}{}^{2}\right)+\left(y_{i}{}^{2}-y_{k}{}^{2}\right)+\left(z_{i}{}^{2}-z_{k}{}^{2}\right)+\left(t_{k}{}^{2}-t_{i}{}^{2}\right)v^{2}\right))\\ =0 \end{array}$$

If we put *k*_1_ = −1 and *k*_2_ = +1 in Equation (11) and simplify it, we get the following equation:

(12) $$\begin{array}{ll} 2\left(x_{k}-x_{j}\right)x+ & 2\left(y_{k}-y_{j}\right)y+2\left(z_{k}-z_{j}\right)z+2\left(t_{j}-t_{k}\right)v^{2}\;t\\ & +\left(\left(x_{j}{}^{2}-x_{k}{}^{2}\right)+\left(y_{j}{}^{2}-y_{k}{}^{2}\right)+\left(z_{j}{}^{2}-z_{k}{}^{2}\right)+\left(t_{k}{}^{2}-t_{j}{}^{2}\right)v^{2}\right)=0 \end{array}$$

Equation (12) is the equation of hyperplane *π_jk_*. This shows that the equation of a third intersection hyperplane *π_jk_* is a linear combination of intersection hyperplanes *π_ij_* and *π_ik_*. Based on Lemma 2, the intersection hyperplanes of each of three sensing hypercones that are constructed from the sensed information of a target object by three sensor nodes makes a pencil.

***Definition 6.*** We define the equations of hyperplanes *π_ij_* and *π_ik_* as *the base of* hyperplane equation *π_jk_* if the equation of a hyperplane *π_jk_* is a *linear combination of* (*dependent on*) hyperplanes equations *π_ij_*, *π_ik_* and denote it as follows:

(13) $$\left(\pi_{\mathrm{ij}},\;\pi_{\mathrm{ik}}\right)\to \pi_{\mathrm{jk}}$$

Our points of interest that give the spatio-temporal information of a target object are located on the intersection points of 4-dimensional degree two intersection surfaces of each pair of sensing hypercones. Computing the intersection of three sensing hypercones of target localization is a difficult work and needs heavy computation. Theorem 2 states that the intersection of three sensing hypercones resides on the axis plane of the pencil that is constructed from the intersection hyperplanes of sensing information. Computing the axis plane is easy and finding the intersection of sensing hypercones with this plane is easier than computing the intersection of three sensing hypercones.

### 4.5. Demonstrating Geometric Properties of Three Sensor Node’s Information

In this part we demonstrate the simulations results of Theorem 2 about properties of sensing information of three sensor nodes 1, 2 and 3 that were introduced in Part 4.2. Figure 5a,b,c shows the three intersection hyperplanes and the three intersection surfaces for sensing hypercones of sensor nodes 1, 2 and 3 in three different points in time. In these figures the intersection hyperplanes are shown in the form of planes and the axis plane is shown in the form of a dotted line at different points in time. The figures show that the intersection hyperplanes make a pencil and displays the intersection surfaces as circles that meet each other in two different points on axis plane of this pencil at three different points in time.

In 4-dimensional space, the intersection surface of each pair of sensing hypercones reside on a hyperplane and three pair of three sensing hypercones meet each other on a curve that resides on the intersection plane of the pencil that these sensing hypercones make. Figure 5d summarizes Figure 5a,b.c and shows that the intersection hyperplanes make a pencil whose axis plane passes through a target object’s spatio-temporal information.

***Definition 7***. Three sensing nodes *i,j* and *k* make a pencil whose axis plane intersects with the intersection of three sensing hypercones on a 2-dimensional curve that we call it *the intersection curve* and denote it by *χ_ijk_*.

**Figure 6.**
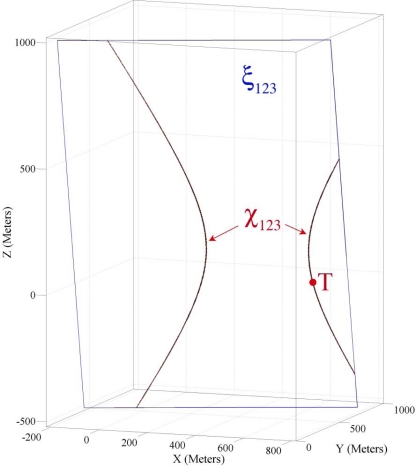
The axis plane of sensor nodes 1, 2 and 3, alongside their intersection curve, in the range of [−4,4] seconds.

If we consider all figures in Figure 5 sequentially and try to figure out the 4-dimensional shape that the intersection surfaces of each triple sensing hypercones make, we see that they make a hyperbola like the one shown in Figure 6. In other words, if we draw these two intersection points of three sensing hypercones in different points in time, it will appear as in Figure 6. This hyperbola lies on the axis plane of the pencil that is made from the triple paired combination of sensing hypercones. This figure shows the axis plane and the intersection curve of three sensing information in the time range of [−4,4].

### 4.6. Geometric Properties of Four Sensor Nodes’ Information

***Lemma 3.*** Three hyperplanes that are not in the same pencil and do not parallel intersect in a straight line.

***Proof.*** Let us assume a set of simultaneous equations constructed from equations of three hyperplanes. If equations of three hyperplanes do not satisfy the condition of Equation (9) and are not parallel, we conclude that equations of these three hyperplanes are linearly independent. The number of unknown parameters is four and the rank of coefficient matrix is three. We can thus conclude that the rank of result will be one and three hyperplanes intersect in a straight line.

***Definition 8.*** Three planes in ℝ^3^ can meet each other in a common point and make a *bundle of planes* if their equations are linearly independent [38].

Now we extend the definition of bundle of planes to ℝ^4^.

***Definition 9.*** All hyperplanes in ℝ^4^ that pass through a common line form a *bundle of hyperplanes*. Any three hyperplanes that are not in the same pencil can be used for computing that line [37]. We name the common line of a bundle of hyperplanes that is formed by the intersection hyperplanes of sensing hypercones *η_i_*, *η_j_*, *η_k_* and *η_l_* as *the axis line* and denote it by *λ_ijkl_*.

***Lemma 4.*** A set of *n* hyperplanes in 4-dimensional space meet each other in a common line and constitute a bundle of hyperplanes if equations of only three hyperplanes of this set are linearly independent.

***Proof.*** Let us assume a set of simultaneous equations of *n* hyperplanes. If the coefficients of only three hyperplanes are linearly independent, the number of unknowns is four and the rank of coefficient matrix is three, therefore the rank of answer is one and they will intersect in a common line.

***Theorem 3.*** The intersection hyperplanes of four sensing hypercones makes a bundle of hyperplanes.

***Proof.*** Four sensing hypercones can have four triple combinations. We prove that non common intersection hyperplanes of each triple combination of four sensing hypercones make a bundle of hyperplanes with non common intersection hyperplanes of other triple combination of sensing hypercones. This proves that all intersection hyperplanes pass through a common line. Now we prove that four different pencils that can be constructed from triple combination of sensing information of four sensing nodes intersect with each other in a common line.

Each pair of triple combinations of four sensing hypercones will have two common hypercones. Let us assume two pencils *ξ_ijk_* and *ξ_ijl_*. The pencil *ξ_ijl_* consists of three intersection hyperplanes *π_ij_*, *π_il_*, and *π_jl_*, and the pencil *ξ_ijk_* consists of three intersection hyperplanes *π_ij_*, *π_ik_*, and *π_jk_*. Each pair of these pencils has a common intersection hyperplane. The common intersection hyperplane of the pencil *ξ_ijk_* and *ξ_ijl_* is the *π_ij_* hyperplane. We prove that the intersection hyperplanes *π_il_* and *π_jl_* from the pencil *ξ_ijl_* make a bundle of hyperplanes with the intersection hyperplanes *π_ik_* and *π_jk_* of the pencil *ξ_ijk_*. The equations of the intersection hyperplanes *π_il_*, *π_jl_* and *π_ik_* are as follows, respectively:

(14) $$\{\begin{array}{l} \pi_{\mathrm{il}}:2\left(x_{l}-x_{i}\right)x+2\left(y_{l}-y_{i}\right)y+2\left(z_{l}-z_{i}\right)z+2\left(t_{i}-t_{l}\right)v^{2}\;t+\\ \left(\left(x_{i}{}^{2}-x_{l}{}^{2}\right)+\left(y_{i}{}^{2}-y_{l}{}^{2}\right)+\left(z_{i}{}^{2}-z_{l}{}^{2}\right)+\left(t_{l}{}^{2}-t_{i}{}^{2}\right)v^{2}\right)=0\\ \pi_{\mathrm{jl}}:2\left(x_{l}-x_{j}\right)x+2\left(y_{l}-y_{j}\right)y+2\left(z_{l}-z_{j}\right)z+2\left(t_{j}-t_{l}\right)v^{2}\;t+\\ \left(\left(x_{j}{}^{2}-x_{l}{}^{2}\right)+\left(y_{j}{}^{2}-y_{l}{}^{2}\right)+\left(z_{j}{}^{2}-z_{l}{}^{2}\right)+\left(t_{l}{}^{2}-t_{j}{}^{2}\right)v^{2}\right)=0\\ \pi_{\mathrm{jk}}:2\left(x_{k}-x_{i}\right)x+2\left(y_{k}-y_{i}\right)y+2\left(z_{k}-z_{i}\right)z+2\left(t_{i}-t_{k}\right)v^{2}\;t+\\ \left(\left(x_{i}{}^{2}-x_{k}{}^{2}\right)+\left(y_{i}{}^{2}-y_{k}{}^{2}\right)+\left(z_{i}{}^{2}-z_{k}{}^{2}\right)+\left(t_{k}{}^{2}-t_{i}{}^{2}\right)v^{2}\right)=0 \end{array}$$

We now prove that the equations of these three hyperplanes are linearly dependent. We write the equations of hyperplanes in Equation (14) as follows:

(15) $$\begin{array}{c} \lambda .\left(A_{\mathrm{il}}\;x+B_{\mathrm{il}}\;y+C_{\mathrm{il}}\;z+D_{\mathrm{il}}\;t+E_{\mathrm{il}}\right)+\mu .\left(A_{\mathrm{jl}}\;x+B_{\mathrm{jl}}\;y+C_{\mathrm{jl}}\;z+D_{\mathrm{jl}}\;t+E_{\mathrm{jl}}\right)\\ +\;\upsilon .\left(A_{\mathrm{ik}}\;x+B_{\mathrm{ik}}\;y+C_{\mathrm{ik}}\;z+D_{\mathrm{ik}}\;t+E_{\mathrm{ik}}\right)=0 \end{array}$$

By setting *λ* *=* −1, *μ =* +1, and *υ =* +1 the above relation is simplified to the following form:

(16) $$\begin{array}{l} -1\times \left(2\left(x_{j}-x_{i}\right)x+2\left(y_{j}-y_{i}\right)y+2\left(z_{j}-z_{i}\right)z+2\left(t_{i}-t_{j}\right)v^{2}\;t\right)+(-1)\\ \;\;\;\;\;\;\;\;\;\;\;\;\;\;\;\;\;\;\;\;\;\;\;\;\;\;\;\times \;\;\left(\left(x_{i}{}^{2}-x_{j}{}^{2}\right)+\left(y_{i}{}^{2}-y_{j}{}^{2}\right)+\left(z_{i}{}^{2}-z_{j}{}^{2}\right)+\left(t_{j}{}^{2}-t_{i}{}^{2}\right)v^{2}\right)\\ \;\;\;\;\;\;\;\;\;\;\;\;\;\;\;\;\;\;\;\;\;\;\;\;\;\;+2\left(x_{k}-x_{i}\right)x+2\left(y_{k}-y_{i}\right)y+2\left(z_{k}-z_{i}\right)z+2\left(t_{i}-t_{k}\right)v^{2}\;t\\ \;\;\;\;\;\;\;\;\;\;\;\;\;\;\;\;\;\;\;\;\;\;\;\;\;\;+\left(\left(x_{i}{}^{2}-x_{k}{}^{2}\right)+\left(y_{i}{}^{2}-y_{k}{}^{2}\right)+\left(z_{i}{}^{2}-z_{k}{}^{2}\right)+\left(t_{k}{}^{2}-t_{i}{}^{2}\right)v^{2}\right)=0 \end{array}$$

We can write Equation (16) in the following simplified form:

(17) $$-\left(A_{\mathrm{ij}}\;x+B_{\mathrm{ij}}\;y+C_{\mathrm{ij}}\;z+D_{\mathrm{ij}}\right)+\left(A_{\mathrm{ik}}\;x+B_{\mathrm{ik}}\;y+C_{\mathrm{ik}}\;z+D_{\mathrm{ik}}\right)=0$$

Equation (17) is a linear combination of hyperplanes *π_ij_* and *π_ik_* equations.

**Figure 7.**
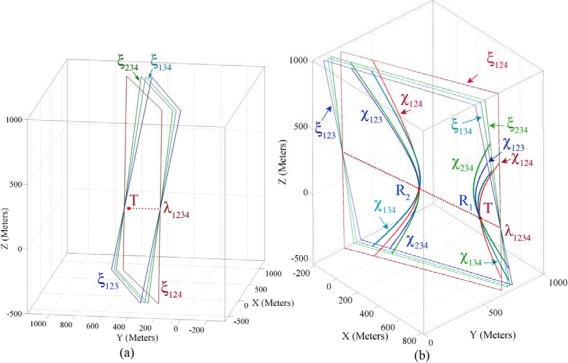
(a). Triple combination of four different sensing nodes’ information making a bundle of hyperplanes that pass through an axis line. (b). Intersection curves of four sensing nodes passing from two different points on the axis line of a bundle of intersection hyperplanes in the time range of [−4,4].

We have proved in Part 4.4 that hyperplanes *π_ij_*, *π_ik_*, and *π_jk_* make a pencil. We know that Equation (17) is like Equation (12) and showed that after simplification we get the equation of *π_jk_* hyperplane in Equation (17). Therefore, from four hyperplanes *π_ij_*, *π_jl_*, *π_ik_*, *π_jk_* three of them are independent. Based on the Lemma 3 and Lemma 4 these four hyperplanes make a bundle of hyperplanes. With a similar reasoning we can prove that each non common intersection hyperplanes of two pencils makes a bundle of hyperplanes and that all these bundles of hyperplanes share a common line like the one shown in Figure 7a.

We can present our proof in other words. Let us assume four sensing hypercones of sensing nodes *i*, *j*, *k* and *l*. These four sensing hypercones have six different paired combinations whose intersection hyperplanes are *π_ij_*, *π_ik_*, *π_il_*, *π_jk_*, *π_jl_*, *π_kl_* In Theorem 2 we proved that in a general form the two intersection hyperplanes of each triple combination of three sensing hypercones are linearly independent and the equation of the third intersection hyperplane from this set is always linearly dependent on the equations of two other intersection hyperplanes. The equations of hyperplanes *π_ij_*, *π_ik_*, *π_il_* are linearly independent because each one uses the equation of a new hypercone. According to Theorem 2 we have:

(18) $$\left(\pi_{\mathrm{ij}},\;\pi_{\mathrm{ik}}\right)\to \pi_{\mathrm{jk}},\;\left(\pi_{\mathrm{ij}},\;\pi_{\mathrm{il}}\right)\to \pi_{\mathrm{jl}},\;\left(\pi_{\mathrm{ik}},\;\pi_{\mathrm{il}}\right)\to \pi_{\mathrm{kl}}$$

Therefore, from the equations of the six intersection hyperplanes of four sensing hypercones, only three of them are linearly independent. Dimension of solution of these simultaneous equations of four intersection hypercones is one and shows that they will intersect in a common line.

Based on Theorem 3, we can find the axis line of a bundle of hyperplanes that are formed by the intersection hyperplanes of four sensing nodes; the axis line intersects with all sensing hypercones only in two common points. We can compute the intersection of the axis line with one of the sensing hypercones for computing the spatio-temporal information of a target object. This computation has lower overhead in comparison to computing the intersections of four sensing hypercones.

### 4.7. Demonstrating the Geometric Properties of Four Sensor Node’s Information

In this part we use the sensing information of the example introduced in Part 4.2 for demonstrating the properties of four sensing hypercones proven in Theorem 3. In Theorem 2 and Part 4.5 we proved and showed that each triple combination of sensing nodes’ information makes a pencil on whose axis plane resides the spatio-temporal information of a target object. Theorem 3 proved that four axis planes of intersection hyperplanes’ pencil meet each other in a common line and all intersection hyperplanes make a bundle of hyperplanes. Figure 7a shows four axis planes passing through a common axis line, where this line passes through a 4-dimensional point that represents the spatio-temporal information of the target object.

Figure 7b shows the axis planes and the intersection curves of four sensing nodes. This figure shows that four intersection curves intersect with each other in two different points *R*_1_ and *R*_2_. Both of these points lie on the axis line of the intersection hyperplanes. Our target localization equations are degree two; therefore we have *two dual mathematically correct answers*. One of these two points is the *feasible* spatio-temporal information of the target object and another answer is *infeasible*. The feasible spatio-temporal information of the target object is shown by point *T* in Figure 7b. To apply Theorem 3, we first compute the axis line and then compute its intersection with one of the sensing hypercones. We call this method *the four coverage axis line* (FCAL) and formulate it in Part 5.1. In Part 4.8 we explain a method of detecting the feasible answer between two dual computed answers.

### 4.8. Elimination of the False Positive Answer of 3-Dimensional Acoustic Target Localization

**Figure 8.**
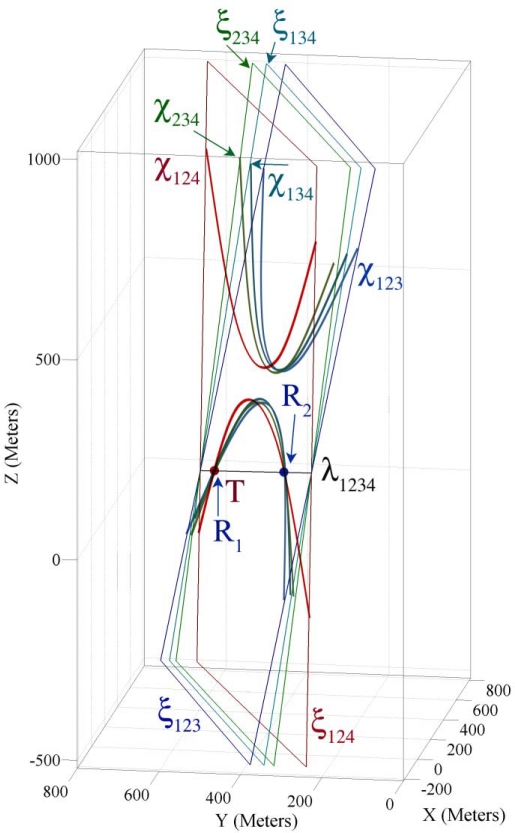
Pitfalls of the FCAL method in computing the accurate spatio-temporal information of a target object when both answers are related to past time.

FCAL method produces two different answers both of which are mathematically correct but only one of them is the feasible 4-dimensional spatio-temporal information of the target object and the other answer is infeasible. We declare a simple method called the *time test* for recognizing the feasible answer. In Theorem 3 we proved that the axis line passes from two intersection points of four sensing hypercones. Computed answers sometimes belong to the up nappe of sound propagation hypercones of Figure 2 and therefore related to the future time. The inherent property of the problem refuses that both answers become related to the future time. We can categories answers as follows:

- *Case I:* If the time of one of the answers say *R*_1_ is before the times of the sound sensing by all four sensor nodes and the time of the other answer, *R*_2_ is after the time of the sound sensing of at least one of the four sensing nodes, answer *R*_1_ is related to past time and answer *R*_2_ is related to future time and is the infeasible answer. An example of case I was shown in Figure 7b.
- *Case II:* If the times of both computed answers *R*_1_ and *R*_2_ is before the reported times of sound sensing by all sensor nodes, both answers are related to the past and time test cannot help the FCAL method to detect the feasible spatio-temporal information of a target object. Figure 8 shows this case which demonstrates the pitfall of four degree sensing coverage and the FCAL method. In this case we can randomly select one of the answers or we cans or refuse to report any answer. Another approach is to report both answers and assign a 50% confidence degree to each answer. In simulation of this method in Part 5.3, if a redundant set of simultaneous equations with sensing information of different set of sensor nodes is constructed, the application of majority voter increases the probability of selecting the feasible answer. This is because it is probable that other set of simultaneous equations do not fall in case II.
- *Case III:* If the axis line is tangent to sound broadcasting hypercone of target object intersects with it on a single point, both answers *R*_1_ and *R*_2_ are the same and both of them are the feasible spatio-temporal information of a target object. The time test method is successful in cases I and III but it cannot detect the correct answer in case II.

We proved that *four degree sensing coverage* *only sometimes* *can determine the accurate spatio-temporal information of a target object in 3-dimensional acoustic target localization.* This fact is independent of the target localization method we use and it is true in Bayesian filtering methods too. Proof of this fact using the Kalman filtering or the Particle filtering is very difficult while the combination of the algebraic and geometric methods is a straightforward method for proving this fact.

## 5. A Proposed Four Sensing Coverage Based Method

### 5.1. Four Coverage Axis Line Method for 3-Dimensional Acoustic Target Localization

Based on Theorem 3 we propose a simple combined algebraic and geometry based method, we called it Four Coverage Axis Line (FCAL) method in Part 4.7. Using properties of Theorem 3 we do not use heavy computations for computing the intersection of four sensing hypercones; instead in the first step we compute the axis line of four sensing hypercones and then we compute its intersection with one of the sensing hypercones. FCAL converts degree two systems of four simultaneous equations to a simple degree one system of three simultaneous equations and greatly decreases the computation overhead.

The simultaneous equations in Equation (1) representing the classic target localization problem are degree two and by differencing these equations, as in following equation, we can eliminate the degree two factors:

(19) $$\{\begin{array}{l} \pi_{12}:{\left(x-x_{1}\right)}^{2}+{\left(y-y_{1}\right)}^{2}+{\left(z-z_{1}\right)}^{2}-\left({\left(x-x_{2}\right)}^{2}+{\left(y-y_{2}\right)}^{2}+{\left(z-z_{2}\right)}^{2}\right)=\\ \left({\left(t_{1}-t\right)}^{2}-{\left(t_{2}-t\right)}^{2}\right)v^{2}\\ \pi_{13}:{\left(x-x_{1}\right)}^{2}+{\left(y-y_{1}\right)}^{2}+{\left(z-z_{1}\right)}^{2}-\left({\left(x-x_{3}\right)}^{2}+{\left(y-y_{3}\right)}^{2}+{\left(z-z_{3}\right)}^{2}\right)=\\ \left({\left(t_{1}-t\right)}^{2}-{\left(t_{3}-t\right)}^{2}\right)v^{2}\\ \pi_{14}:{\left(x-x_{1}\right)}^{2}+{\left(y-y_{1}\right)}^{2}+{\left(z-z_{1}\right)}^{2}-\left({\left(x-x_{4}\right)}^{2}+{\left(y-y_{4}\right)}^{2}+{\left(z-z_{4}\right)}^{2}\right)=\\ \left({\left(t_{1}-t\right)}^{2}-{\left(t_{4}-t\right)}^{2}\right)v^{2} \end{array}$$

If we simplify the above equations we get the equations of intersection hyperplanes as follows:

(20) $$\{\begin{array}{l} \pi_{12}:2\left(x_{2}-x_{1}\right)x+2\left(y_{2}-y_{1}\right)y+2\left(z_{2}-z_{1}\right)z=\\ 2\left(t_{2}-t_{1}\right)v^{2}t+\left(t_{1}{}^{2}-t_{2}{}^{2}\right)v^{2}+\left(x_{2}{}^{2}-x_{1}{}^{2}\right)+\left(y_{2}{}^{2}-y_{1}{}^{2}\right)+\left(z_{2}{}^{2}-z_{1}{}^{2}\right)\\ \pi_{13}:2\left(x_{3}-x_{1}\right)x+2\left(y_{3}-y_{1}\right)y+2\left(z_{3}-z_{1}\right)z=\\ 2\left(t_{3}-t_{1}\right)v^{2}t+\left(t_{1}{}^{2}-t_{3}{}^{2}\right)v^{2}+\left(x_{3}{}^{2}-x_{1}{}^{2}\right)+\left(y_{3}{}^{2}-y_{1}{}^{2}\right)+\left(z_{3}{}^{2}-z_{1}{}^{2}\right)\\ \pi_{14}:2\left(x_{4}-x_{1}\right)x+2\left(y_{4}-y_{1}\right)y+2\left(z_{4}-z_{1}\right)z=\\ 2\left(t_{4}-t_{1}\right)tv^{2}+\left(t_{1}{}^{2}-t_{4}{}^{2}\right)v^{2}+\left(x_{4}{}^{2}-x_{1}{}^{2}\right)+\left(y_{4}{}^{2}-y_{1}{}^{2}\right)+\left(z_{4}{}^{2}-z_{1}{}^{2}\right) \end{array}$$

Equation (20) can be converted into the matrix form as follows:

(21) $$\begin{array}{l} \left[\begin{array}{ccc} 2\left(x_{2}-x_{1}\right) & 2\left(y_{2}-y_{1}\right) & 2\left(z_{2}-z_{1}\right)\\ 2\left(x_{3}-x_{1}\right) & 2\left(y_{3}-y_{1}\right) & 2\left(z_{3}-z_{1}\right)\\ 2\left(x_{4}-x_{1}\right) & 2\left(y_{4}-y_{1}\right) & 2\left(z_{4}-z_{1}\right) \end{array}\right]\;\left[\begin{array}{c} x\\ y\\ z \end{array}\right]\\ \begin{array}{ll} & \;\;\;\;\;\;\;\;\;\;\;\;\;\;\;\;\;\;\;={\left[2\left(t_{2}-t_{1}\right)v^{2}\;2\left(t_{3}-t_{1}\right)v^{2}\;\;\;\;2\left(t_{4}-t_{1}\right)v^{2}\right]}^{T}[t]\\ & \;\;\;\;\;\;\;\;\;\;\;\;\;\;\;\;\;\;\;+\left[\begin{array}{l} \left(t_{1}{}^{2}-t_{2}{}^{2}\right)v^{2}+\left(x_{2}{}^{2}-x_{1}{}^{2}\right)+\left(y_{2}{}^{2}-y_{1}{}^{2}\right)+\left(z_{2}{}^{2}-z_{1}{}^{2}\right)\\ \left(t_{1}{}^{2}-t_{3}{}^{2}\right)v^{2}+\left(x_{3}{}^{2}-x_{1}{}^{2}\right)+\left(y_{3}{}^{2}-y_{1}{}^{2}\right)+\left(z_{3}{}^{2}-z_{1}{}^{2}\right)\\ \left(t_{1}{}^{2}-t_{4}{}^{2}\right)v^{2}+\left(x_{4}{}^{2}-x_{1}{}^{2}\right)+\left(y_{4}{}^{2}-y_{1}{}^{2}\right)+\left(z_{4}{}^{2}-z_{1}{}^{2}\right) \end{array}\right] \end{array} \end{array}$$

Equation (21) can be simplified to:

(22) $$m\;\left[\begin{array}{c} x\\ y\\ z \end{array}\right]=b[t]+c$$

Equation (22) can be solved by using the inverse matrix of *m* as follows:

(23) $$\left[\begin{array}{c} x\\ y\\ z \end{array}\right]=m^{-1}\;b[t]+m^{-1}c$$

Equation (23) has four unknown variables *x,y,z* and *t*, but we have only three simultaneous equations. These simultaneous equations have unlimited answers. Equation (23) yields the equation of the axis line of four sensing hypercones of sensing node’s information. Now we need to compute the intersection of this line with one of the sensing hypercones; the intersection always takes place at two different points. We can use the equation of sensing hypercone of the sensing node *P*_1_ as follows:

(24) $$\eta_{1}:{\left(x-x_{1}\right)}^{2}+{\left(y-y_{1}\right)}^{2}+{\left(z-z_{1}\right)}^{2}-{\left(t_{1}-t\right)}^{2}\;v^{2}=0$$

We represent unknown variables *x*, *y* and *z* in Equation (23) based on the unknown variable *t* and then substitute them in Equation (24) and after simplification we get the following equation:

(25) $$\begin{array}{l} {\left({\left(m^{-1}b\right)}_{1}t+{\left({\left(m^{-1}c\right)}_{1}-x_{1}\right))}^{2}+\left({\left(m^{-1}b\right)}_{2}t+({\left(m^{-1}c\right)}_{2}-y_{1}\right)\right)}^{2}\\ \;\;\;\;\;\;\;\;\;\;\;\;\;\;\;\;\;\;\;\;\;\;\;\;+({\left(m^{-1}b\right)}_{3}\;t+{\left({\left(m^{-1}c\right)}_{3}-z_{1}\right))}^{2}-{\left(t_{1}-t\right)}^{2}\;v^{2}=0 \end{array}$$

Factorizing Equation (25) with respect to *t* yields a degree two equation in the following form:

(26) $$\begin{array}{l} \left({\left(m^{-1}\;b\right)}_{1}{}^{2}+{\left(m^{-1}\;b\right)}_{2}{}^{2}+{\left(m^{-1}\;b\right)}_{3}{}^{2}-v^{2}\right)t^{2}\\ \;\;\;\;\;\;\;\;\;\;\;\;\;\;\;\;\;\;\;\;\;\;\;\;+2({\left(m^{-1}\;b\right)}_{1}\left({\left(m^{-1}\;c\right)}_{1}-x_{1}\right)+{\left(m^{-1}\;b\right)}_{2}\left({\left(m^{-1}\;c\right)}_{2}-y_{1}\right)\\ \;\;\;\;\;\;\;\;\;\;\;\;\;\;\;\;\;\;\;\;\;\;\;\;+{\left(m^{-1}\;b\right)}_{3}\;\left({\left(m^{-1}\;c\right)}_{3}-z_{1}\right)+v^{2}\;t_{1})t+{\left({\left(m^{-1}\;c\right)}_{1}-x_{1}\right)}^{2}\\ \;\;\;\;\;\;\;\;\;\;\;\;\;\;\;\;\;\;\;\;\;\;\;\;+{\left({\left(m^{-1}\;c\right)}_{2}-y_{1}\right)}^{2}+{\left({\left(m^{-1}\;c\right)}_{3}-z_{1}\right)}^{2}-t_{1}{}^{2}\;v^{2}=0 \end{array}$$

Equation (26) is in the form of degree two equation of one variable in the following from:

(27) $${\mathrm{At}}^{2}+\mathrm{Bt}+C=0$$

Equation (27) can be solved by the delta rule method. The inherent structure of the problem causes the delta of Equation (27) never to become negative. Equation (27) gives two different values for variable *t* when delta is greater than zero. Values of *x*, *y*, *z* variables are computed by replacing the computed value of *t* variable in Equation (23). Using time test method that was presented in Part 4.8 we can mostly identify the feasible answer.

### 5.2. Discussion on FCAL Method

Matrix *m* in Equation (22) is *singular* if and only if it is *not reversible* and this happens when the rank of *m* is less than *its dimension* [39]. If the rows of *m* are linearly independent then the rank of *m* is *3* and *m* is *nonsingular* [40,41]. If four sensor nodes *P*_1_, *P*_2_, *P*_3_, and *P*_4_ are located such that no three of them are located on a line, then the matrix *m* will be nonsingular. The probability that three randomly placed sensing nodes are located on a line is very low, though it is not zero. If three sensing nodes are located on a line the system of simultaneous equations of Equation (1) will not have answer. FCAL method has low computational overhead. Computing the answers is not iterative similar to known numerical methods like Newton method. Computation and memory usage of FCAL method at worst and on average are equal to *Θ(1)*.

### 5.3. Simulation Model

For simulative study of the FCAL method we developed and tested the 3-dimensional acoustic target tracking problem in a WSN with randomly distributed wireless sensor nodes. We used the *VisualSense* simulator [42] that builds on and leverages Ptolemy II version 6.0.2 [43,44]. In our simulations we used a single sink node with 40 unique sensor nodes that were spread with normal distribution in a 3-dimensional square field with variation of X position in [0,500] meters range and Y position in [0,500] meters range, and Z position in [0,50] meters range. A target object was rotated ten times in spiral form in this field passing through a unique route in every run such that most areas of simulation field were traversed by all runs. Simulation was run for 400 seconds and a target object regularly broadcasted specific acoustic signals that were detectable by sensor nodes every two seconds. Target localization was carried out 200 times during simulation. The sink node had radio communication radius of 240 meters and other sensor nodes had an equal radio communication and sensing range of 120 meters. We assumed perfect routing without any packet losses and perfect time synchronization with accuracy of 10^−9^ seconds.

### 5.4. Simulation Results

**Figure 9.**
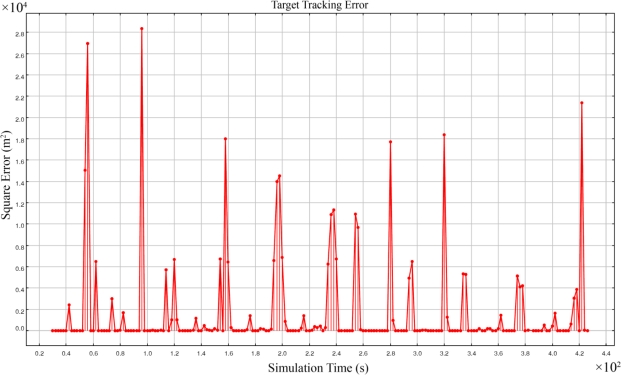
Square error of 3-dimensional acoustic target tracking of FCAL method.

Figure 9 shows the square error of target tracking when we used (1) highly accurate time synchronization with 10^−9^ (seconds) precision, (2) target localization by using the information of only four different sensing nodes in each set of simultaneous equations using the FCAL method, and (3) a simple formal majority voter algorithm. The accuracy of the best time synchronization algorithms in real cases were in the order of 10^−6^ seconds [45,46]. Because our chosen time synchronization accuracy was not attainable in reality, we assumed that our simulations were running under ideal perfect time synchronization. With this very high time synchronization accuracy, the square error was sometimes in the order of 10^4^ m^2^. A variation of formal majority voter presented in [47] was used for fusing the information of target tracking. The mean of spatially distributed 4-dimensional vectors of spatio-temporal information of the target object was computed first. A nearest vector to the mean vector was chosen as a representative. A vector was then randomly selected from a group of candidate vectors whose Euclidian distance was lower than a specific threshold value (similarity parameter) σ from the representative vector; 0.4 was assigned to the σ parameter.

As Figure 9 shows, most of the times the FCAL method computes the accurate spatio-temporal information of the target object. But sometimes reported results had big error values. These outliers happened when case II of FCAL method happened one of the answers was randomly selected and reported. In other words, the source of this big error is the reporting of false positive answers.

### 5.5. Elimination Condition for False Positive Answer

***Definition 10.*** Four hyperplanes that are not parallel and do not belong to a pencil or a bundle of hyperplanes intersect on a point. All hyperplanes passing through the same point form a 3-dimensional extent [37] and we call them *extent of hyperplanes*.

***Theorem 4.*** The intersection hyperplanes of five sensing nodes makes an extent of hyperplanes.

***Proof.*** Let us assume that we have five sensing hypercones *i*, *j*, *k*, *l*, and *m*. Five sensing hypercones have ten different paired combinations whose intersection hyperplanes are π*_ij_*, π*_ik_*, π*_il_*, π*_im_*, π*_jk_*, π*_jl_*, π*_jm_*, π*_kl_*, π*_km_*, and π*_lm_*. In Theorem 2 we proved in a general form that two intersection hyperplanes of each triple combinations of three sensing hypercones are linearly independent and the equations of a third hyperplane always is linearly dependent on the equations of these two hyperplanes. The equations of hyperp lanes π*_ij_*, π*_ik_*, π*_il_* and π*_im_* are linearly independent, because each one uses the equation of different set of sensing hypercones. According to Definition 6 and Theorem 3 we have:

(28) $$\begin{array}{c} \left(\pi_{\mathrm{ij}},\;\pi_{\mathrm{ik}}\right)\to \pi_{\mathrm{jk}},\;\left(\pi_{\mathrm{ij}},\;\pi_{\mathrm{il}}\right)\to \pi_{\mathrm{jl}},\;\left(\pi_{\mathrm{ik}},\;\pi_{\mathrm{il}}\right)\to \pi_{\mathrm{kl}}\\ \left(\pi_{\mathrm{ij}},\;\pi_{\mathrm{im}}\right)\to \pi_{\mathrm{jm}},\;\left(\pi_{\mathrm{il}},\;\pi_{\mathrm{im}}\right)\to \pi_{\mathrm{lm}},\;\left(\pi_{\mathrm{ik}},\;\pi_{\mathrm{im}}\right)\to \pi_{\mathrm{km}} \end{array}$$

Therefore, from equations of ten intersection hyperplanes of five sensing hypercones, only four of them are linearly independent. The equations of the six remaining hyperplanes are linearly dependent on the equations of these four hyperplanes. The system of simulatenous equations of intersection hyperplanes has four unknown variables and four independent equations. Therefore the dimension of answer will be 4 – 4 = 0 implying that the intersection hyperplanes intersect on a common point. This point is our unique and feasible spatio-temporal information of the target object.

***Definition 11.*** We call the common point of an extent of hyperplanes that the intersection hyperplanes of sensing hypercones *η_i_*, *η_j_*, *η_k_*, *η_l_*, and *η_m_*, make as *the extent point* and denote it by *ω_ijklm_*.

**Figure 10.**
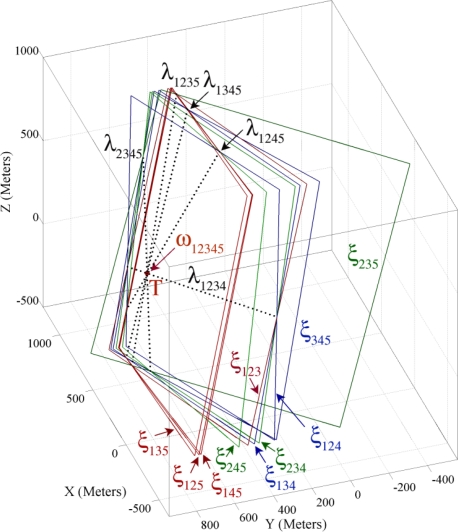
Ten axis planes and five axis lines of their bundle of hyperplanes and their extent point of five sensing nodes.

Let us now assume that we add a fifth sensing node P_5_ (750, 800, 175, 0.7802) to the example given in Part 3.2. Figure 10 shows ten axis planes, five axis lines and the extent point of the intersection hyperplanes. This figure shows that based on Theorem 4 the intersection hyperplanes of five sensing nodes make an extent of hyperplanes. By computing the extent point we can reduce the computation overhead of FCAL method in determining the accurate spatio-temporal information of target object when case II occurs.

## 6. Five Sensing Coverage Proposed Methods

### 6.1. Five Coverage Extent Point Method

We extend the FCAL method and propose the Five Coverage Extent Point (FCEP) method. Based on Theorem 4 we make a system of four simultaneous equations of independent intersection hyperplanes as follows:

(29) $$\{\begin{array}{l} \pi_{12}:2\left(x_{2}-x_{1}\right)x+2\left(y_{2}-y_{1}\right)y+2\left(z_{2}-z_{1}\right)z+2\left(t_{1}-t_{2}\right)v^{2}t=\\ \left(t_{1}{}^{2}-t_{2}{}^{2}\right)v^{2}+\left(x_{2}{}^{2}-x_{1}{}^{2}\right)+\left(y_{2}{}^{2}-y_{1}{}^{2}\right)+\left(z_{2}{}^{2}-z_{1}{}^{2}\right)\\ \pi_{13}:2\left(x_{3}-x_{1}\right)x+2\left(y_{3}-y_{1}\right)y+2\left(z_{3}-z_{1}\right)z+2\left(t_{1}-t_{3}\right)v^{2}t=\\ \left(t_{1}{}^{2}-t_{3}{}^{2}\right)v^{2}+\left(x_{3}{}^{2}-x_{1}{}^{2}\right)+\left(y_{3}{}^{2}-y_{1}{}^{2}\right)+\left(z_{3}{}^{2}-z_{1}{}^{2}\right)\\ \pi_{14}:2\left(x_{4}-x_{1}\right)x+2\left(y_{4}-y_{1}\right)y+2\left(z_{4}-z_{1}\right)z+2\left(t_{1}-t_{4}\right)tv^{2}=\\ \begin{array}{l} \left(t_{1}{}^{2}-t_{4}{}^{2}\right)v^{2}+\left(x_{4}{}^{2}-x_{1}{}^{2}\right)+\left(y_{4}{}^{2}-y_{1}{}^{2}\right)+\left(z_{4}{}^{2}-z_{1}{}^{2}\right)\\ \pi_{15}:2\left(x_{5}-x_{1}\right)x+2\left(y_{5}-y_{1}\right)y+2\left(z_{5}-z_{1}\right)z+2\left(t_{1}-t_{5}\right)tv^{2}=\\ \left(t_{1}{}^{2}-t_{5}{}^{2}\right)v^{2}+\left(x_{5}{}^{2}-x_{1}{}^{2}\right)+\left(y_{5}{}^{2}-y_{1}{}^{2}\right)+\left(z_{5}{}^{2}-z_{1}{}^{2}\right) \end{array} \end{array}$$

We can represent this system of linear equations in the following matrix form:

(30) $$\begin{array}{l} \left[\begin{array}{llll} 2\left(x_{2}-x_{1}\right) & 2\left(y_{2}-y_{1}\right) & 2\left(z_{2}-z_{1}\right) & 2\left(t_{1}-t_{2}\right)v^{2}\\ 2\left(x_{3}-x_{1}\right) & 2\left(y_{3}-y_{1}\right) & 2\left(z_{3}-z_{1}\right) & 2\left(t_{1}-t_{3}\right)v^{2}\\ 2\left(x_{4}-x_{1}\right) & 2\left(y_{4}-y_{1}\right) & 2\left(z_{4}-z_{1}\right) & 2\left(t_{1}-t_{4}\right)v^{2}\\ 2\left(x_{5}-x_{1}\right) & 2\left(y_{5}-y_{1}\right) & 2\left(z_{5}-z_{1}\right) & 2\left(t_{1}-t_{5}\right)v^{2} \end{array}\right]\;\left[\begin{array}{c} x\\ y\\ z\\ t \end{array}\right]\\ \begin{array}{l} \begin{array}{c} \;\;\;\;\;\;\;\;\;\;\;\;\;\;\;\;\;\;\;\;\;\;\;\;\;\;=\left[\begin{array}{l} \left(t_{1}{}^{2}-t_{2}{}^{2}\right)v^{2}+\left(x_{2}{}^{2}-x_{1}{}^{2}\right)+\left(y_{2}{}^{2}-y_{1}{}^{2}\right)+\left(z_{2}{}^{2}-z_{1}{}^{2}\right)\\ \left(t_{1}{}^{2}-t_{3}{}^{2}\right)v^{2}+\left(x_{3}{}^{2}-x_{1}{}^{2}\right)+\left(y_{3}{}^{2}-y_{1}{}^{2}\right)+\left(z_{3}{}^{2}-z_{1}{}^{2}\right)\\ \left(t_{1}{}^{2}-t_{4}{}^{2}\right)v^{2}+\left(x_{4}{}^{2}-x_{1}{}^{2}\right)+\left(y_{4}{}^{2}-y_{1}{}^{2}\right)+\left(z_{4}{}^{2}-z_{1}{}^{2}\right)\\ \left(t_{1}{}^{2}-t_{5}{}^{2}\right)v^{2}+\left(x_{5}{}^{2}-x_{1}{}^{2}\right)+\left(y_{5}{}^{2}-y_{1}{}^{2}\right)+\left(z_{5}{}^{2}-z_{1}{}^{2}\right) \end{array}\right] \end{array} \end{array} \end{array}$$

This method has higher computational cost as it requires the computation of the inverse of a 4 × 4 instead of 3 × 3 matrix of the FCAL method. The cost of computing the inverse of a matrix with dimension *n* is Θ(n^2^) [48]. Most sensor nodes have low computational power and using floating point computations requires more clock cycles, reducing the computation cost when using tiny wireless sensor nodes is essential especially in real time applications.

### 6.2. Five Coverage Extended Axis Line Method

Based on Theorem 4 we propose a second extension to the FCAL method and call this method Five Coverage Extended Axis Line (FCEAL) method. In FCEAL we use the FCAL method for computing the spatio-temporal information of a target object. If case I or case III occurs we select the feasible answer and if case II occurs, we use the sensing information of a fifth sensor node. Only one of the two computed answers satisfies the equation of sensing hypercone of the fifth sensor node.

**Figure 11.**
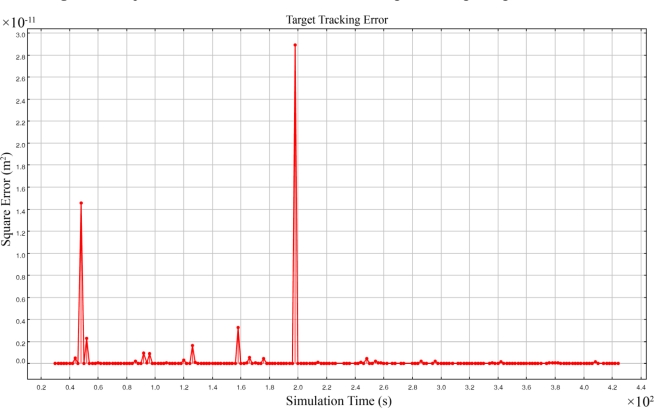
Square error of 3-dimensional acoustic target tracking using FCEP method.

Figure 11 shows the square error of 3-dimensional target tracking using FCEP method. This method in comparison with the FCEP method has less computational overhead because the coefficients matrix of simultaneous linear equations is 3 × 3 and computing its inverse requires less overhead.

FCEP and FCEAL methods produce the same results but only differ in their computation method and computation cost. As Figure 11 shows, the order of square error of results is 10^−11^ m^2^ that is by far smaller than the 10^4^ m^2^ order of the square error of target tracking with four sensing coverage of the FCAL method shown in Figure 9. Simulation results showed that the FCEP and FCEAL methods using five sensing coverage completely eliminates the occurrence of false positive answers.

### 6.3. Five Coverage Redundant Axis Lines Method

Based on Theorem 3 and Theorem 4 we propose a new method relaying on the five degree sensing coverage called Five Coverage Redundant Axis Lines (FCRAL) method as yet another extension to the FCAL method. In FCRAL, each sensor node gathers the sensing information of four neighbor sensing nodes and uses the FCAL method for computation. Two different conditions occur; in case I or case III we can compute accurately the spatio-temporal of target object with 100% confidence degree. However, if case II occurs then the computing node sends both computed answers to sink node with 50% confidence degree. With five sensing coverage, we need at least two groups of sensing information to be constructed, wherein each group has the sensing information of at least four sensor nodes. Five sensing nodes of a target's sound may be located such that some of them cannot communicate with each other in single hop. Therefore, every sensing node sometimes needs to broadcast its sensing information to its neighbor nodes in two hops for making at least two sets of localization simultaneous equations. By this means at least two sets of sensing information of four sensor nodes can be constructed; this must be guaranteed by the management procedures that are enforced on sensor nodes.

**Figure 12.**
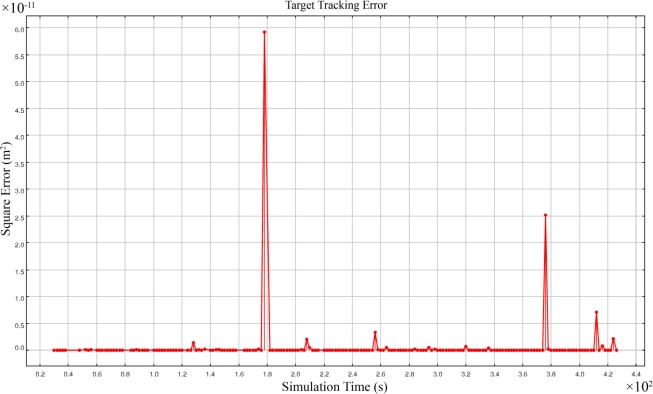
Square error of 3-dimensional target tracking using FCRAL method.

Sensor nodes that are placed in the routing path to the sink node and we call them the fusing nodes, can use a modified formal majority voter algorithm [47,49]. Fusing nodes only send information to the sink node which has 100% confidence level. In worst cases, when all answers received by a fusing node have 50% confidence levels, the fusing node runs a voter algorithm to cluster the received answers and select the answer whose frequency is higher than other answers; the selected answer is then sent with 100% confidence by the fusing node to the sink. For example let us assume that one set contains the sensing information of sensor nodes 1, 2, 3, and 4 and another set contains the sensing information of sensor nodes 2, 3, 4, and 5. If both sets report two answers with 50% confidence levels, we know that both sets must have a common answer which is the feasible spatio-temporal information of a target object and their uncommon answer must differ with each other because a different set of sensing information is used in each set. Therefore the proposed modified formal majority voter selects the feasible answer. Figure 12 shows the square error of simulation results of 3-dimensional acoustic target tracking when we used FCRAL method using the same network set up and scenario mentioned in Part 5.1. The magnitude of the square of error was in the order 10^−11^ m^2^, which is comparatively smaller than the square error of the FCAL method in Figure 9 in Part 5.3 whose magnitude of square error was 10^4^ m^2^ using four sensing coverage.

As Figure 12 shows, we did not have infeasible reported answers and only few outliers existed in the results that were due to the error propagation of computation. Given outliers were rare with no false positive answer we can conclude that 3-dimensional target tracking had a high accuracy.

Figure 11 and Figure 12 show some outliers in the results whose magnitudes are in the scale of 10^−11^ m^2^. As mentioned in Part 5.2, if at least three sensing nodes are located on a straight line then the coefficient matrix *m* is not reversible and the set of simultaneous equations do not have any answer. Similarly, if at least three sensing nodes are such that their locations are near to a straight line, then they are close to being linearly dependent and the determinant of matrix *m* will be close to zero and causes big error propagation in computations. The probability of this condition is very low and the only solution for it is to eliminate these rarely happening outliers from results but we did not eliminate them in this study to clarify that the magnitude of error propagation is comparatively low in comparison with the magnitude of error shown in Figure 9 that was attributed to the error in reporting a false dual answer.

### 6.4. Application of Proposed Methods in Bayesian Filters

Kalman and Particle filters are special types of Bayesian filters that use a measurement model beyond the system model for tracking a target object. Commonly, the measured signals of sensor nodes are used in measurement models [1,2,12]. Measuring the distance of a target object from a sensor node based on the characteristics of the measured acoustic signals in the existence of many different environmental influencing factors and sources of noises is very error prone. Although assumptions of our proposed method require frequent time synchronization and communication between sensor nodes but its accuracy is higher than relying only on the intensity or other features of measured acoustic signals of each individual sensor node. Therefore, our proposed method can be used as part of measurement model in Bayesian filters to provide better estimate of the spatio-temporal information of a target object based on measured acoustic signals.

## 7. Conclusions and Future Work

### 7.1. Conclusions

Given that wireless sensor networks based solutions to 3-dimensional acoustic target tracking with four sensing coverage do not always compute the feasible spatio-temporal information of target objects, we investigated this weakness in a formal setting in this paper. To do so we first combined geometry and algebra for modeling the basics of 3-dimensional acoustic target tracking. These basics are valid for all variations of 3-dimensional acoustic target tracking methods like Bayesian filters. We converted the classic 3-dimensional acoustic target tracking problem to a form combining algebraic and geometric reasoning. This allowed us to study and prove some of the inherent and interesting properties of the problem. Based on these proven properties, we proved that four sensing coverage only under certain conditions guarantees to compute two dual answers, one of which is a feasible answer and another is infeasible answer. We then proved that four sensing coverage does not always guarantee to clarify feasible answer of the 3-dimensional acoustic target localization problem. This was achieved by using a set of lemmas and theorems we proved before applying them to our proposed four coverage axis line (called, FCAL) method for 3-dimensional acoustic target tracking.

We proved that five sensing coverage guarantees to always yield the spatio-temporal information of target objects in 3-dimensional acoustic target tracking. We extended our FCAL method to five sensing coverage in three ways and proposed three methods called five coverage extent point (FCEP) method, five coverage extended axis line (FCEAL) method, and five coverage redundant axis lines (FCRAL) method. We showed that the computational and memory usage overheads of all four methods on average and in the worst cases are equal to *Θ*(1) each. Sources such as bad placement of sensor nodes that caused big error propagation in the proposed methods were discussed too. We also showed that having perfect assumptions and input data are not sufficient conditions for accurate target localization and that paying attention to the mathematical basis of used algorithms is an important issue.

### 7.2. Future work

We did not consider the time synchronization, sensor node localization error, and the sensing and environmental noises in our studies reported in this paper. In real applications though, these factors greatly influence the accuracy and precision of the results. In our previous works the basics of two dimensional target tracking were presented [21] and the error propagation effect of time synchronization error on the results of 2-dimensional target tracking was discussed [50]. Simulative and analytic studies of error propagation of acoustic target localization based on the combination of sensor node localization and time synchronization errors are included in our future work.

The aim of this paper was to prove some facts about the basics of 3-dimensional acoustic target tracking regardless of the above parameters. These facts are valid for all types of 3-dimensional acoustic target tracking methods. Bayesian methods like Kalman filtering and Particle filtering can also use these facts and our proposed methods in their measurement models. Application of the results of this paper to the Bayesian filters in real world applications is currently under investigation by the authors.
